# Using Co-segregation and Loss of Heterozygosity Analysis to Define the Pathogenicity of Unclassified Variants in Hereditary Breast Cancer Patients

**DOI:** 10.3389/fonc.2020.571330

**Published:** 2020-10-02

**Authors:** Rebeca Silveira Grasel, Paula Silva Felicio, André Escremim de Paula, Natalia Campacci, Felipe Antônio de Oliveira Garcia, Edilene Santos de Andrade, Adriane Feijó Evangelista, Gabriela Carvalho Fernandes, Cristina da Silva Sabato, Pedro De Marchi, Cristiano de Pádua Souza, Cláudia Alessandra Andrade de Paula, Giovana Tardin Torrezan, Henrique de Campos Reis Galvão, Dirce Maria Carraro, Edenir Inêz Palmero

**Affiliations:** ^1^Molecular Oncology Research Center, Barretos Cancer Hospital, São Paulo, Brazil; ^2^Center of Molecular Diagnosis, Barretos Cancer Hospital, São Paulo, Brazil; ^3^Department of Medical Oncology, Barretos Cancer Hospital, São Paulo, Brazil; ^4^Oncoclinicas, Rio de Janeiro, Brazil; ^5^Department of Oncogenetics, Barretos Cancer Hospital, São Paulo, Brazil; ^6^Genomic Diagnostic Center, AC Camargo Cancer Center, São Paulo, Brazil; ^7^Genomics and Molecular Biology Group, CIPE - A. C. Camargo Cancer Center, São Paulo, Brazil; ^8^Pele Little Prince Research Institute, Curitiba, Brazil; ^9^Faculdades Pequeno Príncipe, Curitiba, Brazil

**Keywords:** genetics, hereditary breast and ovarian cancer predisposition syndrome, hereditary cancer, variant of unknown significance, segregation analysis

## Abstract

The use of gene panels introduces a new dilemma in the genetics field due to the high frequency of variants of uncertain significance (VUS). The objective of this study was to provide evidence that may help in the classification of these germline variants in terms of their clinical impact and association with the disease in question. A total of 52 unrelated women at-risk for HBOC and negative for *BRCA1/BRCA2* pathogenic variants were evaluated through a gene panel comprising 14 breast and/or ovarian cancer susceptibility genes. Of the 453 germline variants identified, 15 variants (classes 3, 4, and 5) in the *ATM, BRIP1, CHEK2, MRE11A, MUTHY, PALB2, RAD50*, and *RAD51C* genes were evaluated via databases, co-segregation studies and loss of heterozygosity in the tumor. The co-segregation analysis allowed the establishment of an association with the presence of variants and the risk of cancer for variant c.316C>T in the *BRIP1* gene. Four variants of uncertain significance showed loss of heterozygosity in the tumor (*ATM* c.4709T>C; *CHEK2* c.1036C>T; *PALB2* c.1001A>G, and *RAD50* c.281T>C), which is an indication of pathogenicity. Thus, the present study provides novel evidence that favors the association of variants in moderate-risk genes with the development of hereditary breast cancer.

## Introduction

Germline pathogenic *BRCA1* or *BRCA2* variants are responsible for ~15–25% of the hereditary breast/ovarian cancer (HBOC) cases (1, 2). Other than *BRCA1/BRCA2*, several high and moderate cancer genes have been associated with HBOC. The wide use of gene panels has proven to be extremely useful in clinical practice due to the optimization of both time and cost, and to the identification and monitoring of high-risk families for hereditary breast cancer, harboring pathogenic germline variants in genes other than *BRCA1* and *BRCA2*. However, the use of gene panels introduces a new dilemma: a high frequency of variants of uncertain significance (VUS).

In order to assist in the classification of variants, the *American College of Medical Genetics and Genomics* (ACMG) published, in 2015, a report with the *Association for Molecular Pathology* (AMP) and *College of American Pathologists* (CAP) experts' describing the recommendations for germline variants classification, identified through genetic testing (3). This guideline describes the classification of variants based on evidences, such as population, *in silico*, functional and segregation analysis. Among all the criteria, it is worth noting that co-segregation and loss of heterozygosity analysis have been widely used and have an important role in the classification of variants of tumor suppressor genes identified in high risk families (4–7).

In this context, the objective of this study was to evaluate the mutational profile of high- and moderate-risk genes in patients with a personal and/or family history suggestive of HBOC and negative for mutations in the *BRCA1* and *BRCA2* genes and also to provide evidence that may help to classify the identified germline variants in terms of their clinical impact and their association with the disease in question.

## Materials and Methods

### Patients

A retrospective cohort study of 52 unrelated women over 18 years of age with a personal and/or family history (FH) suggestive of HBOC were included. These women were referred to the Department of Cancer Genetics of the Barretos Cancer Hospital (BCH) and tested negative for pathogenic germline variants of the *BRCA1, BRCA2*, and *TP53* genes [as described by Fernandes et al. (8)]. Clinical-pathological data and family history data were collected from the patients' clinical records and the family records in the Department of Cancer Genetics of BCH.

### Molecular Analyses

Genomic DNA obtained from peripheral blood was extracted using the *QIAamp Blood DNA Mini Kit* (Qiagen) according to the manufacturer's instructions. The extracted samples were quantified by fluorimetry (Qubit Fluorometer, Thermo Fisher Scientific). The libraries were prepared using the Ion Ampliseq Library 2.0 kit (Applied Biosystems) according to the manufacturer's protocol. The gene panel used in this study consisted of 14 genes (Supplementary Table 1) related to increased risk (moderate to high) of hereditary breast and/or ovarian cancer. The coding region, along with the immediately adjacent intronic regions (5 bases at the beginning and end of each exon) of each gene, were sequenced using the Ion Torrent PGM platform (Applied Biosystems) following the manufacturer's instructions.

The primary analysis (signal processing, base calling, sequence filtering and mapping) of the generated data was performed using the Torrent suite software, v2.2. The sequences generated were mapped to the reference genome hg19 (*Homo sapiens*). The variants were called and annotated using the Ion Reporter v5.6 software, and the standard parameters suggested by the manufacturer were applied. The first filters for the selection of variants of the gene panel were as follows: exclusion of variants with coverage below 50 X; intronic variants with distance higher than ten base pairs upstream and downstream of exons; variants located in non-coding UTR regions and synonymous variants located in regions not associated with splicing. Copy Number Variations (CNVs) were not assessed by our gene panel NGS approach.

### Data Ansalysis

Besides, after application of the quality filters, all variants were evaluated for their frequency in international population databases [gnomAD (9)] and in a Brazilian database (10) that were used for manually excluding population-specific variants (MAF ≤ 0.01 were maintained) (3). The identified genetic variants were evaluated for their pathogenicity in the ClinVar database (11) and the Human Gene Mutation Database (HGMD) (12). Furthermore, the variants were classified according to the criteria recommended by the ACMG (Varsome web platform) (3). Additionally, the following *in silico* prediction tools were used: PolyPhen-2 (13); MutationTaster (14); Align GVGD (15); SIFT (16); Panther (17); Human Splice Finder (18); Revel (19) CADD (20) and PROVEAN (21).

### Confirmation and Validation of Results

Conventional sequencing: All variants classified by both ClinVar and ACMG as VUS (class 3), likely pathogenic (class 4), and pathogenic (class 5) were confirmed by conventional sequencing (*sanger*). For this validation, the genomic DNAs were amplified by PCR, purified with the enzyme Exosap-IT (*USB*) and Big Dye X terminator kit (Applied Biosystems) and sequenced bi-directionally using the 3500XL platform (Applied Biosystems).

Segregation analysis: For the co-segregation analysis, all families with class 3 germline variants according to the ACMG or ClinVar classification and confirmed by conventional sequencing were invited to participate. All relatives of the index patient with or without cancer at any age who were willing to participate in the study were tested. Co-segregation with disease was considered positive when the variant was present in all affected relatives, and absent in all unaffected individuals (only individuals with diagnosed breast or ovarian cancer were considered as affected).

Loss of heterozygosity (LOH): Variants classified as class 3 and that had available tumor material were subjected to LOH analysis. LOH analysis was conducted using NGS sequencing. Amplicon libraries were prepared by PCR and sequenced using Ion Torrent Proton Platform according to manufacturer's recommended protocol. Germline variant NGS sequencing allows the identification of possible LOH events, taking into consideration the purity of tumor cells and VAF. Data analysis was performed using Torrent Suite 5.10.1 software, and variants of interest were manually inspected with Integrative Genomics Viewer (IGV) visualization. LOH of a wild-type allele was considered when the variant allele had a frequency of >60%. The VAF cut-off value above 60% is based on the model *VAF* = *100/(% tumor cells* + *2X % normal cells)*, which considers that the deletion of the wild-type allele in hereditary syndromes is an early event, thus present in most/all tumor cells.

### Statistical Analysis

Statistical analysis was performed descriptively. SPSS v.21.0 software was used for data storage and statistical analysis.

### Ethical Aspects

The study was approved by the institution's Ethics Committee (40814115.4.0000.5437). All patients included in the study signed the study's consent form.

## Results

### Characterization of the Samples

A detailed clinical-pathological characterization of the 52 patients included in the study is shown in Table 1. In summary, the mean age at first diagnosis of cancer was 41.9 years (ranging from 21 to 61 years). Forty-four patients had breast cancer only (84.6%), three patients had ovarian cancer (5.8%), two patients had breast and ovarian cancer (3.8%), and, three index cases had a personal diagnosis of breast and another tumor type (5.8%). Seventeen percent of the patients were diagnosed with more than one tumor, and one patient had three primary tumors (kidney, breast and thyroid cancer), the first of which was diagnosed when the patient was 31 years old.

**Table 1:** Clinicopathological characteristics of the 52 patients included in the study.

	***N***	**%**
**Primary tumor site**		
Breast	44	84.6
Ovary	3	5.7
Breast and ovary	2	3.8
Breast and Sarcoma	1	1.9
Kidney, breast and thyroid	1	1.9
Melanoma and breast	1	1.9
**Breast and ovarian cancer**		
No	50	96.1
Yes	2	3.8
**Bilateral breast cancer**		
No	48	92.3
Yes	4	7.6
**Multiple tumors**		
No	43	82.6
Yes	9	17.3
**More than two primary tumors**		
No	51	98.0
Yes	1	1.9
**Histological type**		
Intraductal/*in situ*	4	8.0
Invasive ductal carcinoma	43	86.0
Invasive lobular carcinoma	2	4.0
Metaplastic carcinoma	1	2.0
**Ignored**	3	–
Pathological staging		
*In situ*	4	8.3
IA	6	12.5
IB	2	4.1
IIA	6	12.5
IIB	5	10.4
IIIA	16	33.3
IIIB	9	18.7
Ignored	5	–
**Molecular subtype**		
Luminal A	3	6.0
Luminal B1 - HER2 negative	29	58.0
Luminal B2 - HER2 positive	6	12.0
HER2 super express	6	12.0
Basal-like or TNBC^*^	6	12.0
Ignored	3	–

* *TNBC, Triple negative breast cancer*.

Most of the patients included in the study were from the state of São Paulo (Southeast Region 64.2%), were white (86.3%), married (66.7%), and had completed high school (35.3%)—(Supplementary Table 2). Most participants were pre-menopausal (76.7%) and had first-degree relatives with breast cancer (56.9%); and among the postmenopausal/perimenopausal women, none was undergoing hormone replacement therapy (Supplementary Table 3).

Among breast tumors, the mean age at diagnosis was 42.6 years of age. The predominant histology was invasive ductal carcinoma (86.0%), followed by carcinoma *in situ* (8.0%). Four of them had bilateral breast cancer (7.7%). There was a predominance of clinical and pathological stage IIIA tumors, with 33.3% for both. Regarding molecular subtypes, there was a predominance of luminal B1 tumors (58%), while triple negative and Her2-overexpressing tumors together represented ~24% of cases (Table 1).

Among the five women with a personal history of ovarian cancer, the mean age at diagnosis of ovarian cancer was 43.4 years, ranging from 21 to 60 years. Among the five ovarian cases, the histology was available for four cases (all of them were serous ovarian carcinomas), and the predominant pathological stage was IIIC.

Regarding the family history of cancer, the presence of breast cancer in mother-daughter pairs was observed in 41.2% of cases (cancer at any age). When breast cancer in first-degree relatives before age 50 was evaluated, it was observed that 51% of patients had at least one case in their family. In addition, 23.5% of the patients had at least one family member with multiple tumors (Supplementary Table 4).

### Molecular Characterization

**Figure 1:**
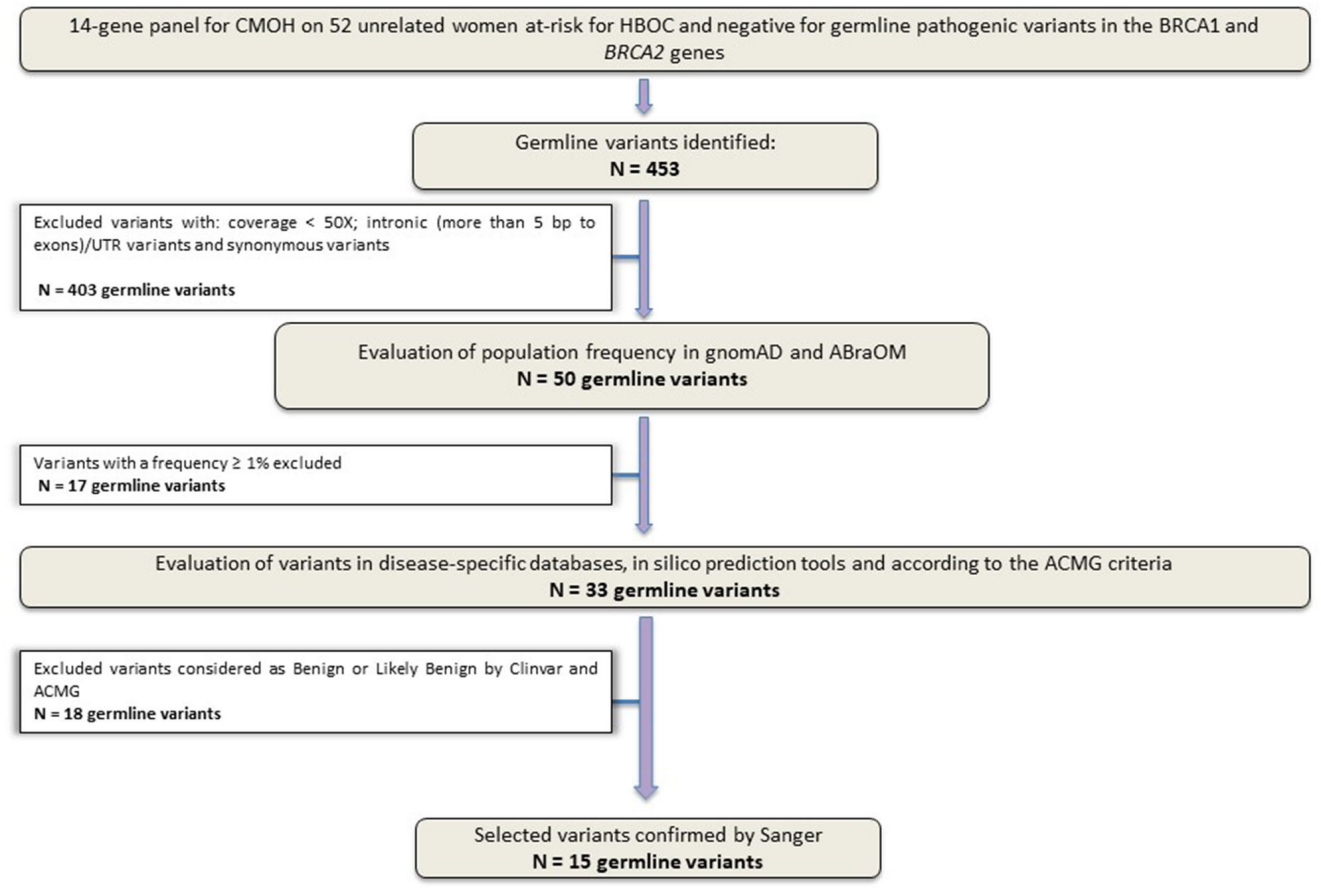
Study design flowchart. Variants selection workflow. Panel sequencing data from 52 unrelated Brazilian women at-risk for HBOC, without germline pathogenic variants in *BRCA1* and *BRCA2* genes.

Sequencing of the 14 genes identified 453 variants. After the application of the quality filters, 50 variants were maintained for the following classification phases. Subsequently, other 35 variants were excluded: (1) variants with a population frequency ≥1% and (2) variants reported as Benign/Probably Benign by ClinVar or according to the ACMG classification. The flowchart shown in Figure 1 details the filters applied to germline variants prioritization employed in this study.

After applying all filters described above, a total of 15 unique variants were identified. Two loss of function variants were considered Pathogenic/Probably Pathogenic by the ACMG and ClinVar: a frameshift variant in *MUTYH* (in heterozygosis): c.1147delC and the splicing variant c.315-2A>G in the *MRE11A* gene, respectively. VUS were considered to be all those classified by ClinVar or by the ACMG as class 3, and 13 variants were found in 11 women (21%). Table 2 contains all information from the databases used and the *in silico* predictions of the 15 germline variants identified.

**Table 2:** Description of the 15 variants identified in the 14-gene panel.

	**Germinative variant identification**			**Pathogenicity classification (classes 1 to 5)**			**Population frequency**		***In silico predictions***								
				**ACMG and disease-specific databases**			**Frequency In %**		**Computational simulations of pathogenicity**								
**Gene**	**Patient ID**	**cDNA**	**Protein**	**ACMG**	**ClinVar**	**HGMD**	**ABraOM**	**gnomAD**	**Align GVGD**	**Panther**	**Hum. Spl. Finder**	**SIFT**	**PolyPhen**	**Mut.Tast**	**PROVEAN**	**CADD**	**REVEL**
*ATM*	85	c.1810C>T	p.Pro604Ser	3	CI	DC	0.00821	0.00422	C0	NR	AS	T	PD	DC	N	24.3	0.415
*ATM*	133	c.4709T>C^*^	p.Val1570Ala	2	CI	VUS	0.00164	0.00045	C0	NR	NI	T	B	Pol	N	8.67	0.152
*ATM*	1046	c.9086G>A^*^	p.Gly3029Asp	3	CI	NR	NR	0.00012	C65	NR	AS	I	B	DC	N	15.8	0.230
*BRIP1*	306	c.316C>T^*^	p.Arg106Cys	2	CI	NR	NR	0.00010	NR	B	AS	T	B	Pol	N	22.5	0.067
*CHEK2*	1095	c.1036C>T^*^	p.Arg346Cys	3	VUS	NR	NR	0.00005	C65	PD	AS	I	PD	DC	D	33	0.780
*CHEK2*	1209	c.1312G>T	p.Asp438Tyr	3	CI	VUS	NR	0.00039	C25	PD	AS	I	PD	DC	D	33	0.337
*MRE11A*	1209	c.315-2A>G	.	5	PP	NR	NR	0.00000	NR	NR	AS	NP	NP	NP	NP	NP	NP
*MRE11A*	133	c.482A>G^*^	p.Lys161Arg	3	VUS	NR	NR	0.00001	C0	PD	AS	T	B	DC	N	12.8	0.342
*MUTYH*	974	c.1147delC	p.Ala385fs	5	P	DC	NR	0.00006	NR	NR	AS	NP	NP	NP	NP	NP	NP
*MUTYH*	755	c.1267C>T	p.Arg423Cys	3	CI	DC	NR	0.00083	NR	NR	NP	I	PD	Pol	N	22.9	0.615
*PALB2*	85	c.1001A>G	p.Tyr334Cys	2	CI	NR	NR	0.00010	C0	B	AS	T	B	Pol	D	0.06	0.014
*RAD50*	869	c.1397A>C	p.Gln466Pro	3	VUS	NR	NR	0.00000	C25	PD	NI	I	B	DC	N	19.6	0.131
*RAD50*	274	c.281T>C	p.lle94Thr	3	VUS	NR	NR	0.00004	C25	B	NI	T	B	DC	D	20.9	0.153
*RAD51C*	640	c.1009G>T^*^	p.Val337Leu	3	NR	NR	NR	NR	NR	NR	NI	T	B	DC	N	22.5	0.190
*RAD51C*	598	c.244C>T	p.His82Tyr	3	VUS	NR	NR	NR	NR	PB	NI	T	B	Pol	N	4.63	0.033

* *Germline variants that have segregation study; ACMG: 1, Benign; 2, Probably Benign; 3, VUS; 4, Probably Pathogenic; 5, Pathogenic; CI, Conflicting Interpretations; VUS, Variant of Uncertain Significance; PP, Probably Pathogenic; P, Pathogenic; DC, Disease Causing; AS, Affect Splicing; NI, no impact on splicing; T, Tolerant; I, Intolerant; NP, Not Predicted; PD, Possibly Damaging; B, Benign; N, Neutral; D, Deleterious; PB, Probably Benign; NR, not reported; Pol, Polymorphism*.

*Clinvar, date of last consult: June, 4th, 2020*.

**Figure 2:**
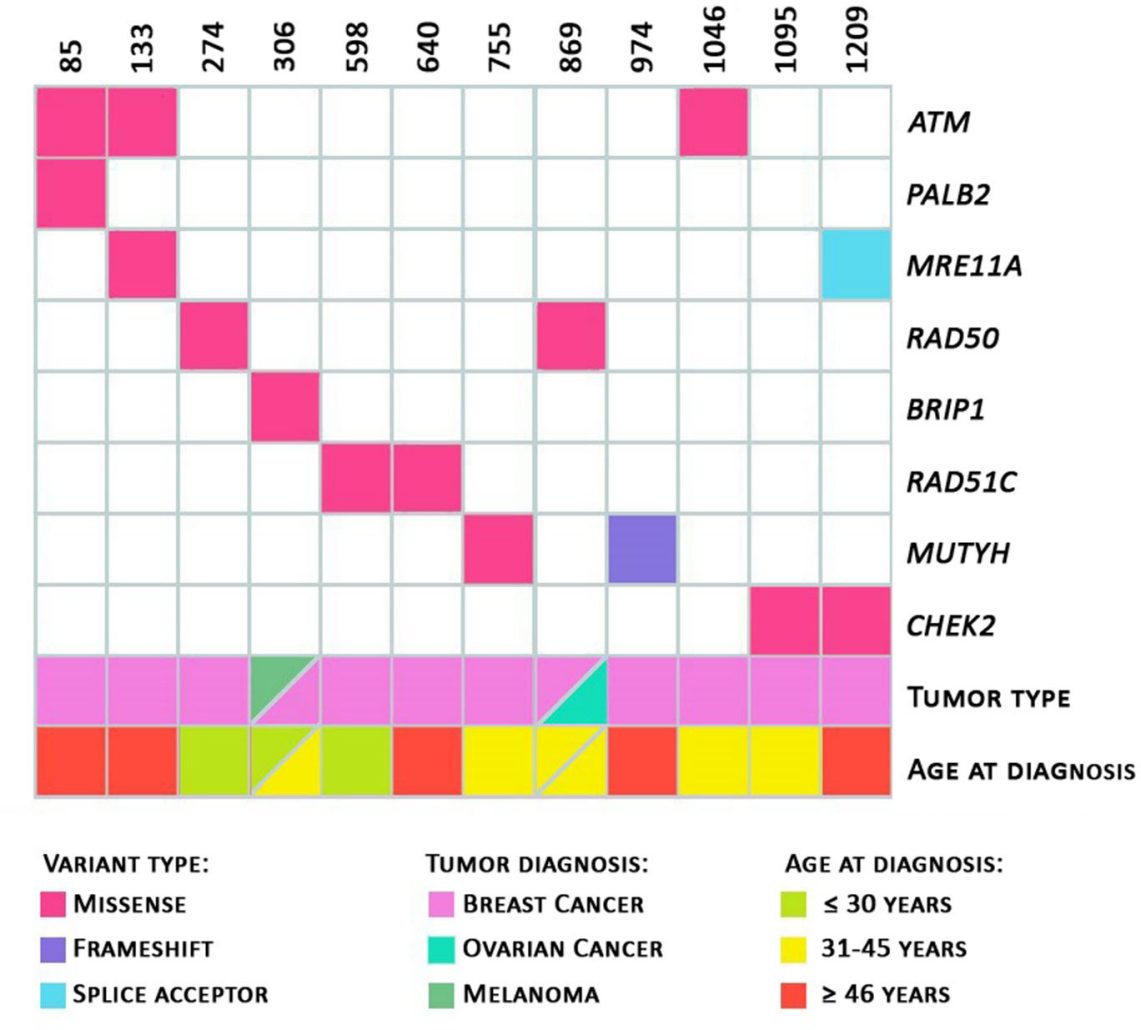
Heatmap. Total number of variants per patient. Variants identified with information regarding the tumor diagnosed and the patient's age at diagnosis; and age at diagnosis (in green: diagnosis ≤ 30 years of age; in yellow: 31 to 45 years of age; in red: ≥ 46 years of age) are represented.

Regarding the personal history of cancer of the 12 probands (with identified class 3, 4, or 5 variants), as shown in Figure 2, seven patients (58.3%) were diagnosed with cancer before the age of 45 and three were diagnosed before the age of 30. Two patients, ID306 (presence of the variant in the *BRIP1* gene, c.316C>T) and ID869 (presence of variant in the *RAD50* c.1397A>C gene) had two primary tumors: melanoma and breast, and breast and ovary, respectively. The patients with pathogenic germline variants in the *MUTYH* and *MRE11A* genes had unilateral breast tumors diagnosed after 46 years of age.

### Segregation and LOH Analysis

**Figure 3:**
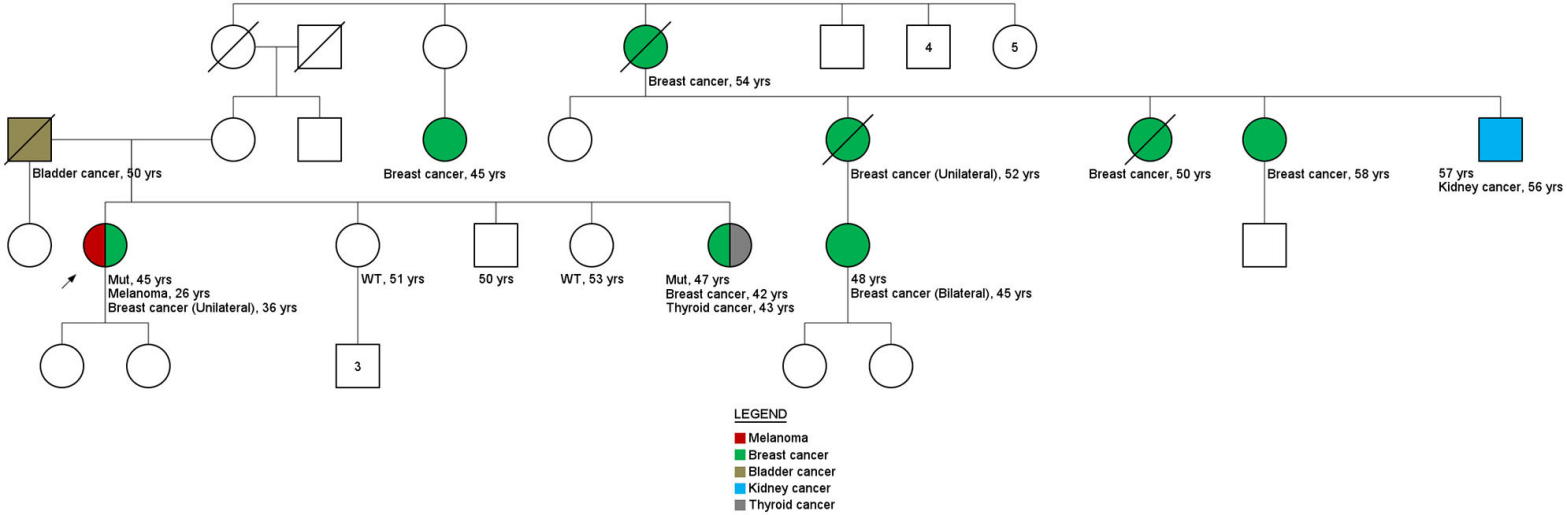
Family history. Pedigree family ID306. Variant c.316C>T in the *BRIP1* gene.

Of the 15 germline variants resulting from the gene panel, it was possible to perform a more in-depth analysis of the biological-clinical impact in 9 of them. For six variants (five families), it was possible to perform co-segregation analysis. In addition, the LOH analysis was performed for seven VUS, and both analyses—co-segregation and LOH—were performed for four variants (Table 3, Supplementary Figure 1). The variant c.4709C>T in the *ATM* gene (class 2 according to the ACMG and conflicting data according to ClinVar) was included in the analysis because it was present in the same patient (ID133) as one *MRE11A* class 3 variant. Pedigree of index cases, where segregation and/or LOH analysis were performed, are depicted in Supplementary Figures 2–7 and in Figure 3.

**Table 3:** Segregation and LOH analysis.

					**Cancer-Free**		**Cancer-affected**		
**ID Patient**	**GENE**	**c.**	**p.**	**Relatives** **(Total)**	**Carrier** **(*n*)**	**Non carrier** **(*n*)**	**Carrier** **(*n*)**	**Non carrier** **(*n*)**	**LOH**
640	*RAD51C*	c.1009G>T	p.Val337Leu	10	5 (60♂; 31♀; 37♀; 65♀; 68♀)	4 (29♂; 38♂; 52♂; 68♀)	0	1 (53♀)	NP
1046	*ATM*	c.9086G>A	p.Gly3029Asp	7	1 (52♀)	5 (61♂; 41♂; 61♀)	0	1 (59♀)	NP
1095	*CHEK2*	c.1036C>T	p.Arg346Cys	5	2 (36♂; 44♂)	2 (50♀; 70♀)	0	1 (42♀)	Yes
306	*BRIP1*	c.316C>T	p.Arg106Cys	3	0	2 (51♀; 53♀)	1 (47♀)	0	No
133	*MRE11A*	c.482A>G	p.Lys161Arg	5	2 (55♀; 65♂)	3 (60♀; 61♀; 67♂;)	0	0	No
133	*ATM*	c.4709T>C	p.Val1570Ala	5	2 (61♀; 65♂)	3 (55♀; 60♀; 67♂)	0	0	Yes
274	*RAD50*	c.281T>C	p.lle94Thr	-	-	-	-	-	Yes
85	*ATM*	c.1810C>T	p.Pro604Ser	-	-	-	-	-	No
85	*PALB2*	c.1001A>G	p.Tyr334Cys	-	-	-	-	-	Yes

*NP, not performed*.

For all variants evaluated through segregation analysis, the sex and the age of the relatives are detailed in Table 3. Given the fact that no cancer affected relatives of family ID133 were available for testing, segregation analysis was not possible. Variants under evaluation were not identified in the cancer-affected relatives tested (1 per family) of ID640, ID1046, and ID1095 families, but were carried by one (ID1046), two (ID1095), and five (ID640) cancer-free relatives (Table 3).

Interestingly, results obtained for variant c.316C>T in *BRIP1* gene of ID306 patient's family (Figure 3) shown that, only the sister's patient with cancer history (breast cancer at 42 years of age and thyroid cancer at 53 years of age) carried the c.316C>T variant (two other siblings without cancer history were tested). These results are an indicative of the of the co-segregation of the variant with the disease.

Regarding the LOH analyses, among the seven VUS evaluated, the loss of the normal allele was identified in four of them: the variants c.1036C>T in the *CHEK2* gene; c.4709T>C variant (*ATM* gene); c.281T>C in the *RAD50* gene, and c.1001A>G in the *PALB2* gene (Supplementary Figure 1).

## Discussion

The patients included in the study were women considered to be at high risk for HBOC. Fifteen germline variants classified as VUS, probably pathogenic and pathogenic were identified in moderate-risk genes (22–24). The frequency of pathogenic germline variants in patients who previously tested negative for pathogenic variants in *BRCA1/BRCA2* was 3.8%. Although it is quite difficult to compare mutation frequencies among studies, due to differences in inclusion criteria and/or ethnicity, our mutation frequency corroborates other studies based on family cancer history inclusion criteria (25–32).

Regarding the pathogenic variants identified, one of them is located in the *MRE11A* gene, which is considered a partner of the *ATM* gene in terms of signaling damage to and repair of double-stranded DNA (33, 34). This gene was described in the first studies using NGS as a gene conferring a moderate risk of HBOC (OR = 2.88, *P* = 0.0090) and therefore was included in panels (34). However, more recent studies with significant sample sizes (case-control) revealed a low risk associated with the *MRE11A* gene. In the classification by Lee et al. (24), which evaluated the clinical validity of genes usually included in panels, the *MRE11A* is classified as “disputed,” i.e., there are conflicting data and/or divergent opinions about its association to HBOC. In our study the index patient who carried the pathogenic germline variant in the *MRE11A* gene was diagnosed with breast cancer at 58 years of age, and her mother had an ovarian tumor diagnosed after 50 years of age. Her family had three generations affected by cancer and a total of 11 individuals with cancer. In addition to the pathogenic germline variant in the *MRE11A* gene, this patient also had a VUS in the *CHEK2* c.1312G>T gene. Southey et al. (35) showed evidence of the relationship of this VUS (*CHEK2* c.1312G>T) with risk of prostate cancer, with OR 2.21 (95% CI 1.06 to 4.63, *p* = 0.030), in European men. However, no case of prostate cancer was reported in our family.

The second pathogenic mutation has been identified in the tumor suppressor gene *MUTYH*. Recent studies point to a low risk of cancer in carriers of monoallelic pathogenic variants in *MUTYH* in HBOC (22, 36). In the present study, the heterozygous *MUTYH* mutation carrier was diagnosed with breast carcinoma at 46 years of age. Additionally, eight cases of breast cancer and two cases of prostate cancer were present in the family history.

Thirteen germline variants of uncertain significance were identified, corresponding to a VUS frequency of 21%. The percentage of VUS, although relatively high, is comparable to that reported in other studies that included moderate and low-risk genes, such as the one published in 2018 by Xie et al. (37), which evaluated 100 women and found a VUS rate of 34.25% in 10 genes associated with susceptibility to breast cancer (excluding *BRCA1* and *BRCA2*).

Among the variants in which the co-segregation analysis could be performed, evidence of an association was found for the germline missense variant c.316C>T in the *BRIP1* gene, present in ID306. Although only three relatives were tested, the family member with a previous diagnosis of breast and thyroid tumors had the variant in question. Nevertheless, it is worth noting that the index patient had two primary tumors: melanoma, diagnosed at 26 years of age, and breast cancer, at 36 years old. The family history suggests that the c.316C>T germline variant in the *BRIP1* gene may be involved in the development of cancer in this family, although the analysis of the tumor material of the index patient did not show LOH. However, loss of the normal allele can also be caused by other genetic and epigenetic processes, such as occurrence of a pathogenic point mutation at the functional allele and promoter hypermethylation.

The presence of pathogenic germline variants in *BRIP1* has been specifically related to ovarian tumors (38, 39). However, other studies have found a moderate association with the risk of breast tumors. A case-control study conducted by Couch et al. (40) showed an OR of 1.27 for breast cancer and 1.67 when both breast cancer and ovarian cancer were included. In the study by Tung and collaborators (36), among 488 patients with breast cancer, four women (0.81%) had a pathogenic variant in the *BRIP1* gene. In the family of patient ID306, no cases of ovarian cancer were reported by the index patient. This fact corroborated the studies by Tung et al. (36) (out of the four patients analyzed, only one had a history of ovarian cancer) and Slavin et al. (23), which identified *BRIP1* mutations in families with other tumors then ovarian. The melanoma phenotype present in patient ID306 and the bladder phenotype present in the patient's father are noteworthy because both were also present in the study by Tung et al. (36). These results suggest that in-depth analyses of the identified variant should continue, together with a close family monitoring and screening.

Regarding the other families in which co-segregation analysis was performed, there was no co-segregation between the variants in *ATM* c.9086G>A, *CHEK2* c.1036C>T, *RAD51C* c.1009G>T, and the disease. However, taking into consideration that only one cancer-affected individual was tested in each family, these findings need to be confirmed in a larger number of relatives. Besides, result needs to be interpreted with caution given the incomplete penetrance of these genes and the moderate risk of cancer associated with them (23, 26, 36, 41–43).

In the family where the co-segregation of the *CHEK2* variant was analyzed, although co-segregation could not be observed, it is worthy to reinforce that this “negative” result needs to be interpreted with caution given the incomplete penetrance of this gene and the fact that LOH was observed in the tumor tissue, suggesting a pathogenic behavior for this variant. In the clinical classification of the relationship of the *CHEK2* gene with HBOC, the association is considered “definitive” for breast tumors (24). Regarding specifically the variant c.1036C>T, it was previously identified by Southey et al. (35) in a case-control study involving breast cancer (42,671 cases and 42,164 controls), prostate cancer (22,301 cases and 22,320 controls), and ovarian cancer (14,542 cases and 23,491 controls), and the results showed strong evidence of an association with the risk of development of breast cancer, with an OR of 5.06 (95% CI 1.09 to 23.5) (35).

For some variants, co-segregation analysis was not possible, but the LOH analysis was performed. The VUS in the gene *PALB2* c.1001A>G, identified in ID85, showed LOH in the tumor. The *PALB2* gene is considered a high-risk gene (40) for hereditary breast cancer. In the study by Slavin et al. (23), the *PALB2* gene had a significant OR of 6.95 (CI 3.71 to 12.70), and in the study by Couch et al. (40), the OR was 7.46 (CI 5,12–11,19). Additionally, in addition to breast cancer, new phenotypes are being associated with the gene, such as pancreatic cancer (42) and gastric cancer (44) (ID85 had two cases of gastric cancer in her family history). Preventive measures and risk management already exist for carriers of the pathogenic germline variant of this gene (https://www.nccn.org/professionals/physician_gls/pdf/genetics_screening.pdf, 2019), which some authors are currently considering “*BRCA3”* (40).

We also identified LOH in the tumor specimen of the patient with the variant c.281T>C (*RAD50* gene). In the most recent studies, the *RAD50* gene demonstrated a low OR for breast cancer. In the study by Couch et al. (40), an OR = 0.77 was observed (CI 0.52-1.61), and in the study by Slavin et al. (23) only one woman had a pathogenic germline variant in this gene. Regarding the “gene-disease” evaluation in the study by Lee et al. (24), it was observed that the classification is considered “limited” for breast tumors and “disputed” for ovarian tumors, once again suggesting that the risk of cancer associated with the gene is still uncertain.

Although there are conflicting reports in the literature regarding the management of patients with class 3 germline variants, the guidelines of the Brazilian Society of Medical Genetics (45) and NCCN (46) recommend that individuals with VUS should be managed based on the risk of the gene along with the personal and family history of cancer. Discussing genetic results with patients, especially those with VUS-type variants or pathogenic variants in genes whose risk is not well-founded, is not easy. It is a challenge for clinicians and for their patients, who face decisions about screening and prevention strategies in the case of different outcomes.

The results presented in this study can contribute to the definition of the pathogenicity of VUS in breast/ovarian susceptibility genes, especially for the variants in which some indicative of pathogenicity has been found (co-segregation with the disease and/or LOH). Further validation could be obtained by functional analyses. The present study had several limitations, which may contribute to the relatively low frequency of pathogenic variants identified. Among these, it is worth noting that the number of patients tested was relatively low compared to other recently published studies. Besides, although relatively rare, the presence of CNVs were not evaluated. In addition, as the understanding of the role of moderate-penetrance genes on breast/ovarian cancer susceptibility is constantly being updated, some genes that were recently reported as HBOC-associated were not included in the panel. Finally, due to a limitation in the access to relatives with cancer and to tumor material, the segregation and LOH analysis were not possible in all families with identified VUS.

## Conclusion

Of the 52 unrelated women with a personal and family history suggestive of breast and/or ovarian cancer, two pathogenic germline variants and 13 germline VUS were identified through the use of a gene panel containing 14 genes associated with high- and moderate-risks of breast and ovarian cancer.

The co-segregation analysis in 5 families allowed the establishment of an association with the presence of the variant and the risk of cancer for variant c.316C>T in the *BRIP1* gene. Four variants showed LOH in the tumor specimen (*ATM* c.4709T>C; *CHEK2* c.1036C>T; *PALB2* c.1001A>G, and *RAD50* c.281T>C), which is an indication of pathogenicity. Thus, the present study provides novel evidence that favors the association of variants in moderate-risk genes with the development of hereditary breast cancer.

## Data Availability Statement

The datasets presented in this article are not readily available due to privacy and ethical restrictions. Requests to access the datasets should be directed to the corresponding author.

## Ethics Statement

The studies involving human participants were reviewed and approved by Comitê de Ética em Pesquisa, Hospital de Câncer de Barretos. The patients/participants provided their written informed consent to participate in this study.

## Author Contributions

RG: methodology, formal analysis, investigation, data analysis, data curation, and writing—original draft. PF: formal analysis, investigation, data curation, and writing—original draft. AE, GF, and CS: methodology, investigation, data curation, and writing—review and editing. NC: patient recruitment, data curation, and writing—review and editing. FG, EA, and AE: data analysis; data curation, and writing—review and editing. PD: data analysis; data curation, and writing—original draft. CP and HG: patient recruitment; data curation, and writing—review and editing. CAAP and GT: LOH analysis, data analysis, and writing—review and editing. DC: conceptualization, LOH analysis, data analysis, and writing—review and editing. EP: conceptualization, writing—review and editing, supervision, project administration, and funding acquisition. All authors: contributed to the article and approved the submitted version.

## Conflict of Interest

The authors declare that the research was conducted in the absence of any commercial or financial relationships that could be construed as a potential conflict of interest.

## References

[1] Couch FJ Nathanson KL Offit K. Two decades after BRCA: setting paradigms in personalized cancer care and prevention. Science. (2014) 343:1466–70. 10.1126/science.1251827 24675953 PMC4074902

[2] Nielsen FC van Overeem Hansen T Sorensen CS. Hereditary breast and ovarian cancer: new genes in confined pathways. Nat Rev Cancer. (2016) 16:599–612. 10.1038/nrc.2016.72 27515922

[3] Richards S Aziz N Bale S Bick D Das S Gastier-Foster J. Standards and guidelines for the interpretation of sequence variants: a joint consensus recommendation of the American College of Medical Genetics and Genomics and the Association for Molecular Pathology. Genet Med. (2015) 17:405–24. 10.1038/gim.2015.30 25741868 PMC4544753

[4] Gracia-Aznarez FJ Fernandez V Pita G Peterlongo P Dominguez O de la Hoya M. Whole exome sequencing suggests much of non-BRCA1/BRCA2 familial breast cancer is due to moderate and low penetrance susceptibility alleles. PLoS ONE. (2013) 8:e55681. 10.1371/journal.pone.0055681 23409019 PMC3568132

[5] Diaz-Gay M Franch-Exposito S Arnau-Collell C Park S Supek F Munoz J. Integrated analysis of germline and tumor DNA identifies new candidate genes involved in familial colorectal cancer. Cancers. (2019) 11:3. 10.3390/cancers11030362 30871259 PMC6468873

[6] Duran-Lozano L Montalban G Bonache S Moles-Fernandez A Tenes A Castroviejo-Bermejo M. Alternative transcript imbalance underlying breast cancer susceptibility in a family carrying PALB2 c.3201+5G>T. Breast Cancer Res Treat. (2019) 174:543–50. 10.1007/s10549-018-05094-8 30552643

[7] Pastorino L Andreotti V Dalmasso B Vanni I Ciccarese G Mandala M. Insights into genetic susceptibility to melanoma by gene panel testing: potential pathogenic variants in ACD, ATM, BAP1, and POT1. Cancers. (2020) 12:4. 10.3390/cancers12041007 32325837 PMC7226507

[8] Fernandes GC Michelli RA Galvao HC Paula AE Pereira R Andrade CE. Prevalence of BRCA1/BRCA2 mutations in a Brazilian population sample at-risk for hereditary breast cancer and characterization of its genetic ancestry. Oncotarget. (2016) 7:80465–81. 10.18632/oncotarget.12610 27741520 PMC5348334

[9] Monkol SL Konrad JK Eric VM Kaitlin ES Eric B Timothy F. Analysis of Protein-Coding Genetic Variation in 60,706 Humans. Available online at: http://gnomad.broadinstitute.org/

[10] AbraOM Available online at: http://abraom.ib.usp.br/

[11] COSMIC Catalogue of Somatic Mutations In Cancer Available online at: www.Sanger.ac.uk/genetics/CGP/cosmic

[12] HGMD Human Gene Mutation Database Available online at: www.hgmd.cf.ac.uk

[13] PolyPhen-2 PolyPhen-2 Available online at: www.genetics.bwh.harvard.edu/pph2

[14] MutationTaster Available online at: www.mutationtaster.org

[15] AlignGVGD Align GVGD Available online at: http://agvgd.hci.utah.edu/agvgd_input.php

[16] SIFT Sorting Intolerant from Tolerant Available online at: http://sift.bii.a-star.edu.sg/

[17] Panther Available online at: http://www.pantherdb.org/tips/tips_csnpScores.jsp

[18] Desmet FO Hamroun D Lalande M Collod-Beroud G Claustres M Beroud C. Human Splicing Finder: an online bioinformatics tool to predict splicing signals. Nucleic Acids Res. (2009) 37:e67. 10.1093/nar/gkp215 19339519 PMC2685110

[19] Ioannidis NM Pejaver RJ Middha V McDonnell S Baheti SK Musolf S. REVEL: an ensemble method for predicting the pathogenicity of rare missense variants. Am J Hum Genet. (2016) 99:877–85. 10.1016/j.ajhg.2016.08.016 27666373 PMC5065685

[20] Kircher M Witten DM Jain P O'Roak BJ Cooper GM Shendure J. A general framework for estimating the relative pathogenicity of human genetic variants. Nat Genet. (2014) 46:310–5. 10.1038/ng.2892 24487276 PMC3992975

[21] Choi SY Chan AP. PROVEAN web server: a tool to predict the functional effect of amino acid substitutions and indels. Bioinformatics. (2015) 31:2745–7. 10.1093/bioinformatics/btv195 25851949 PMC4528627

[22] Slavin TP Niell-Swiller M Solomon I Nehoray B Rybak C Blazer KR Clinical Application of Multigene Panels: Challenges of Next-Generation Counseling and Cancer Risk Management. Front Oncol. (2015) 5:208 10.3389/fonc.2015.00271 26484312 PMC4586434

[23] Slavin TP Maxwell KN Lilyquist J Vijai J Neuhausen SL Hart SN The contribution of pathogenic variants in breast cancer susceptibility genes to familial breast cancer risk. NPJ Breast Cancer. (2017) 3:22 10.1038/s41523-017-0046-2 28649662 PMC5466608

[24] Lee K Seifert BA Shimelis H Ghosh R Crowley SB Carter NJ. Clinical validity assessment of genes frequently tested on hereditary breast and ovarian cancer susceptibility sequencing panels. Genet Med. (2018). 10.1038/s41436-018-0361-5 30504931 PMC6579711

[25] Tung N Battelli C Allen B Kaldate R Bhatnagar S Bowles K. Frequency of mutations in individuals with breast cancer referred for BRCA1 and BRCA2 testing using next-generation sequencing with a 25-gene panel. Cancer. (2015) 121:25–33. 10.1002/cncr.29010 25186627

[26] Thompson ER Rowley SM Li N McInerny S Devereux L Wong-Brown MW. Panel testing for familial breast cancer: calibrating the tension between research and clinical care. J Clin Oncol. (2016) 34:1455–9. 10.1200/JCO.2015.63.7454 26786923

[27] Moran O Nikitina D Royer R Poll A Metcalfe K Narod SA. Revisiting breast cancer patients who previously tested negative for BRCA mutations using a 12-gene panel. Breast Cancer Res Treat. (2017) 161:135–42. 10.1007/s10549-016-4038-y 27798748

[28] Weitzel JN Neuhausen SL Adamson A Tao S Ricker C Maoz A. Pathogenic and likely pathogenic variants in PALB2, CHEK2, and other known breast cancer susceptibility genes among 1054 BRCA-negative Hispanics with breast cancer. Cancer. (2019) 125:2829–36. 10.1002/cncr.32083 31206626 PMC7376605

[29] Kaneyasu T Mori S Yamauchi H Ohsumi S Ohno S Aoki D. Prevalence of disease-causing genes in Japanese patients with BRCA1/2-wildtype hereditary breast and ovarian cancer syndrome. NPJ Breast Cancer. (2020) 6:25. 10.1038/s41523-020-0163-1 32566746 PMC7293299

[30] Lerner-Ellis J Sopik V Wong A Lazaro C Narod SA Charames GS. Retesting of women who are negative for a BRCA1 and BRCA2 mutation using a 20-gene panel. J Med Genet. (2020) 57:380–4. 10.1136/jmedgenet-2019-106403 31784482

[31] Velazquez C Esteban-Cardenosa KL Avila Cobos EM Lastra E Abella LE Germline genetic findings which may impact therapeutic decisions in families with a presumed predisposition for hereditary breast and ovarian cancer. Cancers. (2020) 2:8 10.3390/cancers12082151 PMC7465232 32756499

[32] Yoo J Lee GD Kim JH Lee SN Chae H Han E. Clinical validity of next-generation sequencing multi-gene panel testing for detecting pathogenic variants in patients with hereditary breast-ovarian cancer syndrome. Ann Lab Med. (2020) 40:148–54. 10.3343/alm.2020.40.2.148 31650731 PMC6822011

[33] Lavin MF. ATM and the Mre11 complex combine to recognize and signal DNA double-strand breaks. Oncogene. (2007) 26:7749–58. 10.1038/sj.onc.1210880 18066087

[34] Damiola F Pertesi M Oliver J Le Calvez-Kelm F Voegele C Young EL. Rare key functional domain missense substitutions in MRE11A, RAD50, and NBN contribute to breast cancer susceptibility: results from a Breast Cancer Family Registry case-control mutation-screening study. Breast Cancer Res. (2014) 16:R58. 10.1186/bcr3669 24894818 PMC4229874

[35] Southey MC Goldgar DE Winqvist R Pylkas K Couch F Tischkowitz M. PALB2, CHEK2 and ATM rare variants and cancer risk: data from COGS. J Med Genet. (2016) 53:800–11. 10.1136/jmedgenet-2016-103839 27595995 PMC5200636

[36] Tung N Lin NU Kidd J Allen BA Singh N Wenstrup RJ. Frequency of Germline Mutations in 25 Cancer Susceptibility Genes in a Sequential Series of Patients With Breast Cancer. J Clin Oncol. (2016) 34:1460–8. 10.1200/JCO.2015.65.0747 26976419 PMC4872307

[37] Xie Y Li G Chen M Guo X Tang L Luo X. Mutation screening of 10 cancer susceptibility genes in unselected breast cancer patients. Clin Genet. (2018) 93:41–51. 10.1111/cge.13063 28580595

[38] Rafnar T Gudbjartsson DF Sulem P Jonasdottir A Sigurdsson A Jonasdottir A. Mutations in BRIP1 confer high risk of ovarian cancer. Nat Genet. (2011) 43:1104–7. 10.1038/ng.955 21964575

[39] Ramus SJ Song H Dicks E Tyrer JP Rosenthal AN Intermaggio MP. Germline mutations in the BRIP1, BARD1, PALB2, and NBN genes in women with ovarian cancer. J Natl Cancer Inst. (2015) 107:11. 10.1093/jnci/djv214 26315354 PMC4643629

[40] Couch FJ Shimelis H Hu C Hart SN Polley EC Na J. Associations Between Cancer Predisposition Testing Panel Genes and Breast Cancer. JAMA Oncol. (2017) 3:1190–6. 10.1001/jamaoncol.2017.0424 28418444 PMC5599323

[41] Cybulski C Lubinski J Wokolorczyk D Kuzniak W Kashyap A Sopik V. Mutations predisposing to breast cancer in 12 candidate genes in breast cancer patients from Poland. Clin Genet. (2015) 88:366–70. 10.1111/cge.12524 25330149

[42] Hu C Hart SN Bamlet WR Moore RM Nandakumar K Eckloff BW. Prevalence of pathogenic mutations in cancer predisposition genes among pancreatic cancer patients. Cancer Epidemiol Biomarkers Prev. (2016) 25:207–11. 10.1158/1055-9965.EPI-15-0455 26483394 PMC4754121

[43] Schubert S van Luttikhuizen JL Auber B Schmidt G Hofmann W Penkert J The identification of pathogenic variants in BRCA1/2 negative, high risk, hereditary breast and/or ovarian cancer patients: High frequency of FANCM pathogenic variants. Int J Cancer. (2018) 144:2683–94. 10.1002/ijc.31992 30426508

[44] Fewings E Larionov A Redman J Goldgraben MA Scarth J Richardson S. Germline pathogenic variants in PALB2 and other cancer-predisposing genes in families with hereditary diffuse gastric cancer without CDH1 mutation: a whole-exome sequencing study. Lancet Gastroenterol Hepatol. (2018) 3:489–98. 10.1016/S2468-1253(18)30079-7 29706558 PMC5992580

[45] Sociedade Brasileira de Genética Médica (2019). Available online at: https://diretrizes.amb.org.br/_BibliotecaAntiga/cancer-familial.pdf

[46] NCCN Guidelines (2019). Available online at: https://www.nccn.org/professionals/physician_gls/pdf/genetics_screening.pdf

