# Supplementary material for: Using Co-segregation and Loss of Heterozygosity Analysis to Define the Pathogenicity of Unclassified Variants in Hereditary Breast Cancer Patients
Source: Front Oncol. 2020 Oct 2;10:571330. doi: 10.3389/fonc.2020.571330 (PMC7566163; doi:10.3389/fonc.2020.571330)
Supplement: Supplementary file 1 [file Data_Sheet_1.docx]

Supplementary Material

# Supplementary Tables

**Table S1.** Gene Panel

| **Gene** | **Seq Ref Panel** | **Gene** | **Seq Ref Panel** |
| --- | --- | --- | --- |
| ***ATM*** | NM_000051.3 | ***PALB2*** | NM_024675.3 |
| ***BARD1*** | NM_000465.3 | ***RAD50*** | NM_005732.3 |
| ***BRIP1*** | NM_032043.2 | ***RAD51C*** | NM_058216.2 |
| ***CHEK2*** | NM_001005735.1 | ***TP53*** | NM_000546.5 |
| ***MRE11A*** | NM_005591.3 | ***CDH1*** | NM_004360.3 |
| ***MUTYH*** | NM_001128425.1 | ***PTEN*** | NM_000314.4 |
| ***NBN*** | NM_002485.4 | ***STK11*** | NM_000455.4 |

**Table S2.** Sociodemographic data of the 52 women included in the study

| **Sociodemographic data** | **N** | **%** |
| --- | --- | --- |
| **Self-declared color** |  |  |
| White | 44 | 86.3 |
| Brown | 6 | 11.8 |
| Black | 1 | 2 |
| Ignored | 1 | - |
| **Scholarity** |  |  |
| Illiterate/incomplete elementary school | 8 | 15.7 |
| Complete primary education | 9 | 17.6 |
| Complete high school | 18 | 35.3 |
| Complete higher education | 16 | 31.4 |
| Ignored | 1 | - |
| **Marital Status** |  |  |
| Not married | 11 | 21.6 |
| Married/stable union | 34 | 66.7 |
| Widower | 2 | 3.9 |
| Divorced | 4 | 7.8 |
| Ignored | 1 | - |
| **Profession** |  |  |
| Does not work | 11 | 22 |
| Works without a formal contract | 34 | 68 |
| Works with a formal contract | 2 | 4 |
| Housewife | 3 | 6 |
| Ignored | 2 | - |
| **Origin** |  |  |
| DF | 1 | 1.9 |
| ES | 1 | 1.9 |
| GO | 2 | 3.8 |
| MG | 7 | 13.2 |
| PE | 1 | 1.9 |
| PR | 3 | 5.7 |
| RO | 1 | 1.9 |
| RR | 1 | 1.9 |
| SP | 34 | 64.2 |
| TO | 1 | 1.9 |
| **Place of birth (state)** |  |  |
| BA | 2 | 3.8 |
| DF | 1 | 1.9 |
| GO | 2 | 3.8 |
| MG | 8 | 15.4 |
| MS | 1 | 1.9 |
| MT | 1 | 1.9 |
| PR | 4 | 7.7 |
| SP | 32 | 61.5 |
| Ignored | 1 | - |

DF: Distrito Federal; GO: Goias; MG: Minas Gerais; MS: Mato Grosso do Sul; PE: Pernambuco; PR: Paraná; RO: Rondônia; RR: Roraima; SP: São Paulo; TO: Tocantins; BA: Bahia; MT: Mato Grosso.

**Table S3.** Variables related to risk of developing breast and / or ovarian cancer (n = 52)

| **Risk variables for CMOH** | **N** | **%** |
| --- | --- | --- |
| **Smoking** |  |  |
| No | 38 | 79.2 |
| Yes | 10 | 20.8 |
| Ignored | 4 | - |
| **Menopausal status:** |  |  |
| Pré | 33 | 76.7 |
| Peri | 1 | 2.3 |
| Pós | 9 | 20.9 |
| Ignored | 9 | - |
| **HRT** |  |  |
| No | 9 | 100 |
| Yes | 0 | 0 |
| Ignored | 43 | - |
| **First degree relative with breast CA** |  |  |
| No | 22 | 43.1 |
| Yes | 29 | 56.9 |
| Ignored | 1 | - |

HRT: Hormone replacement therapy; CA: cancer

**Table S4.** Details of the proband´s family history (n=51)

| **Family History** |  | **N (%)** |
| --- | --- | --- |
| **Mother with breast cancer** | No | 30 (58.8%) |
|  | Yes | 21 (41.2%) |
| **Presence of ovarian cancer at any age** | No | 37 (72.5%) |
|  | Yes | 14 (27.5%) |
| **Presence of Mother and Daughter / Pairs of Sisters with Breast Cancer <50 years** | No | 25 (49.0%) |
|  | Yes | 26 (50.9%) |
| **Presence of Mother and Daughter / Pairs of Sisters with Ovarian Cancer** | No | 48 (94.1%) |
|  | Yes | 3 (5.8%) |
| **Presence of Mother and Daughter / Pairs of Sisters with Breast and / or Ovarian Cancer** | No | 45 (88.2%) |
|  | Yes | 6 (11.8%) |
| **Presence of prostate cancer** | No | 35 (68.6%) |
|  | Yes | 16 (31.4%) |
| **Presence of pancreatic cancer** | No | 49 (96.1%) |
|  | Yes | 2 (3.9%) |
| **Presence of colorectal cancer** | No | 33 (64.7%) |
|  | Yes | 18 (35.3%) |
| **Presence of thyroid cancer** | No | 47 (92.1%) |
|  | Yes | 4 (7.8%) |
| **Presence of gastric cancer** | No | 44 (86.3%) |
|  | Yes | 7 (13.7%) |
| **Presence of endometrial cancer** | No | 49 (96.0%) |
|  | Yes | 2 (3.9%) |
| **Presence of sarcoma** | No | 46 (90.2%) |
|  | Yes | 5 (9.8%) |
| **Presence of adrenocortical cancer** | No | 50 (98.0%) |
|  | Yes | 1 (2.0%) |
| **Presence of CNS Cancer** | No | 50 (98.0%) |
|  | Yes | 1 (2.0%) |
| **Presence of individuals with multiple tumors** | No | 39 (76,5%) |
|  | Yes | 12 (23,5%) |
|  |  |  |

CNS: central nervous system.

# Supplementary Figures


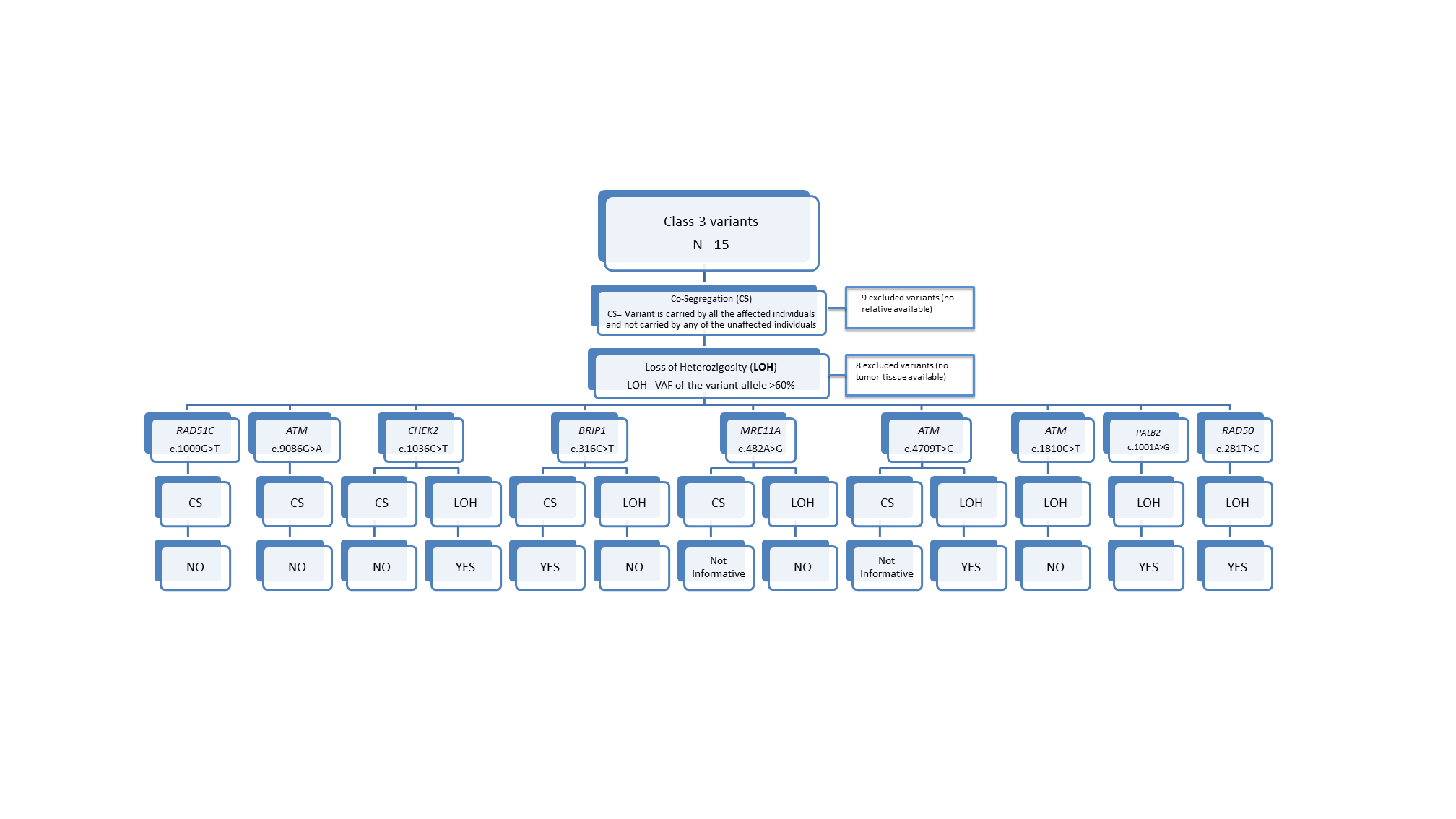


**Supplementary Figure 1.** Flowchart illustrating the criteria and variants submitted to co-segregation and/or LOH analysis. (CS=co-segregation; LOH=loss of heterozygosity)


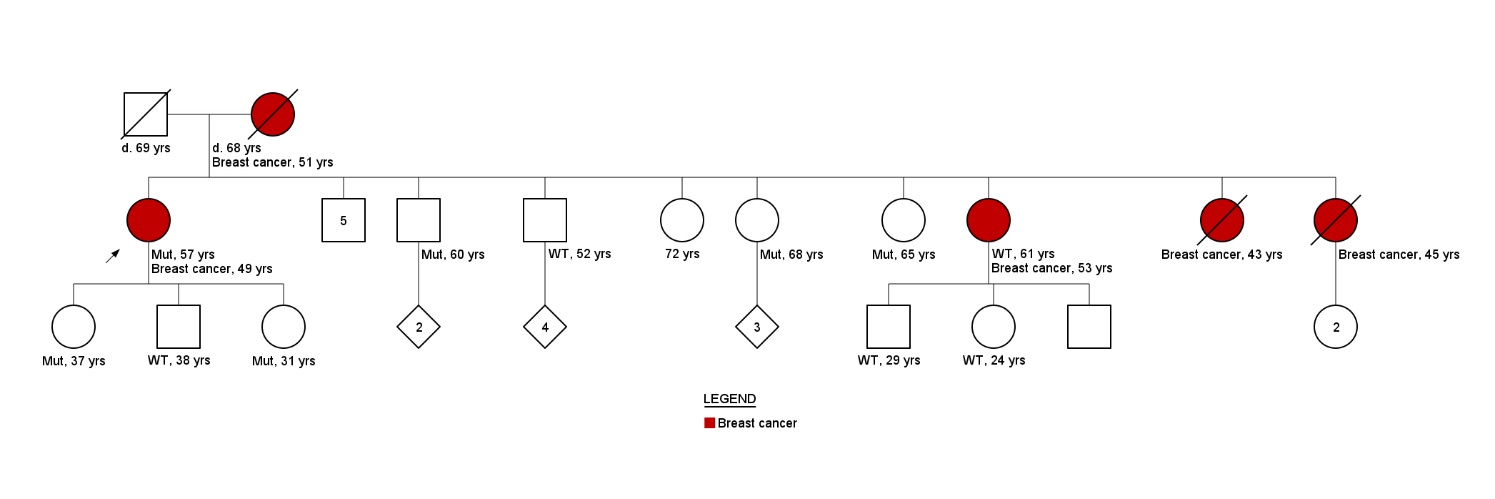


**Supplementary Figure 2**: Family history. Pedigree family ID640. Variant c.1009G>T in the *RAD51C*.


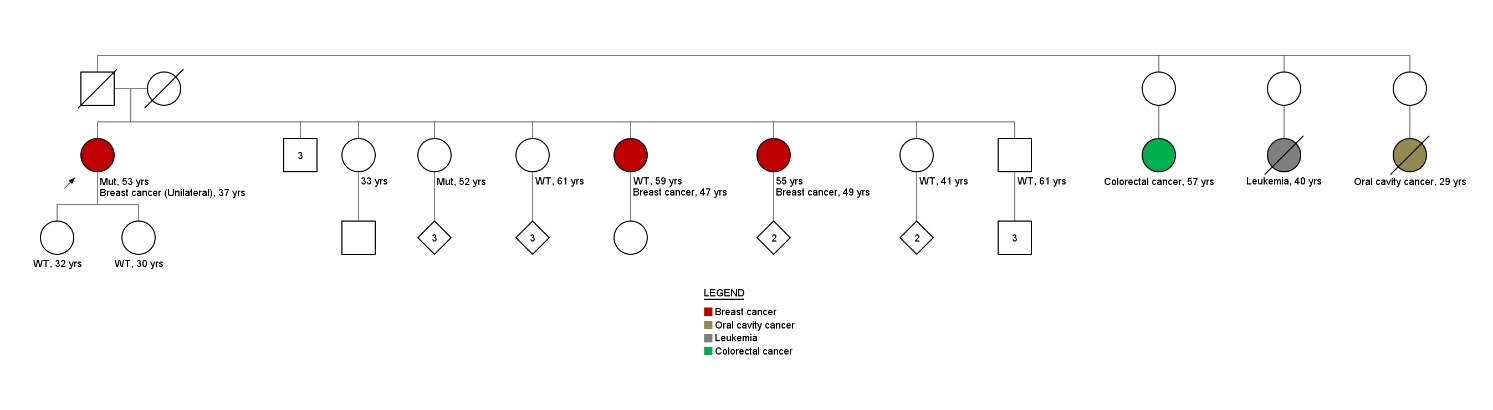


**Supplementary Figure 3:** Family history. Pedigree family ID1046. Variant c.9086G>A in the *ATM.*


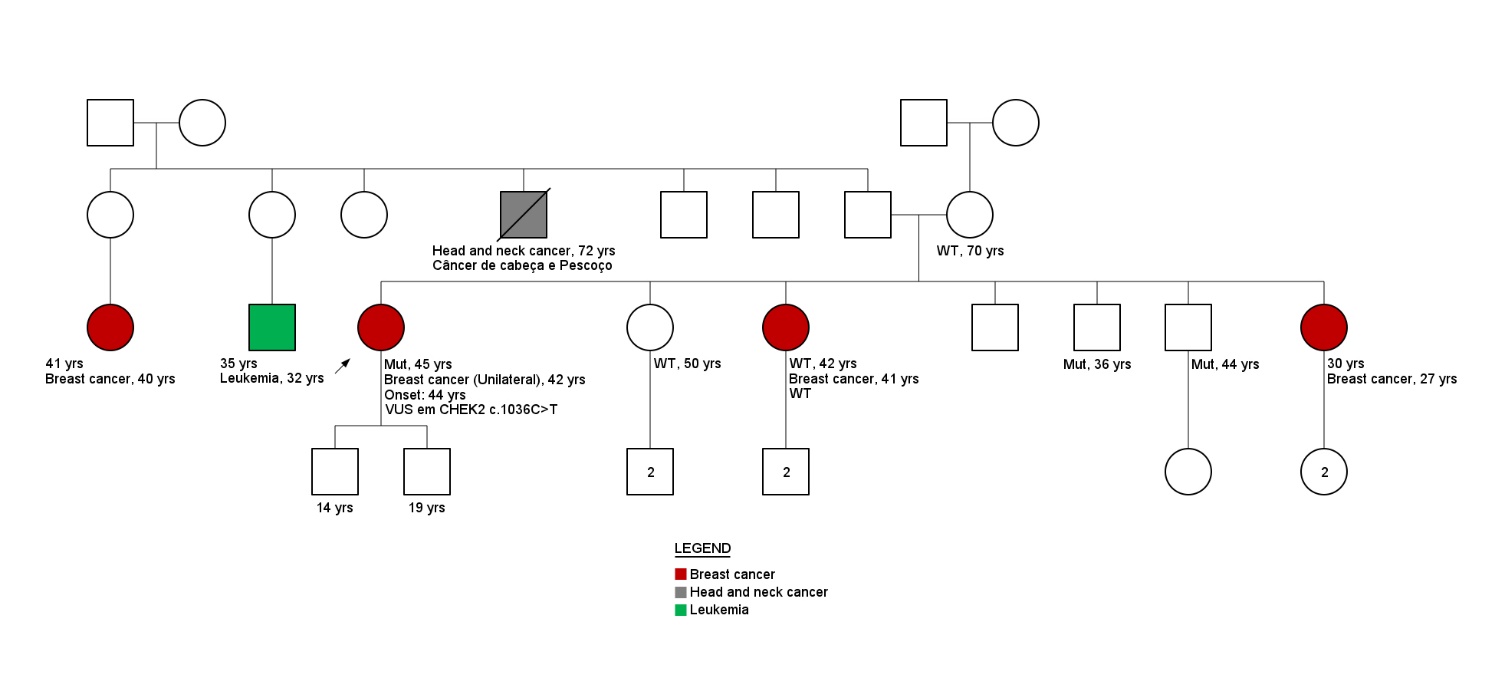


**Supplementary Figure 4:** Family history. Pedigree family ID1095. Variant c.1036C>T in the *CHEK2.*

*
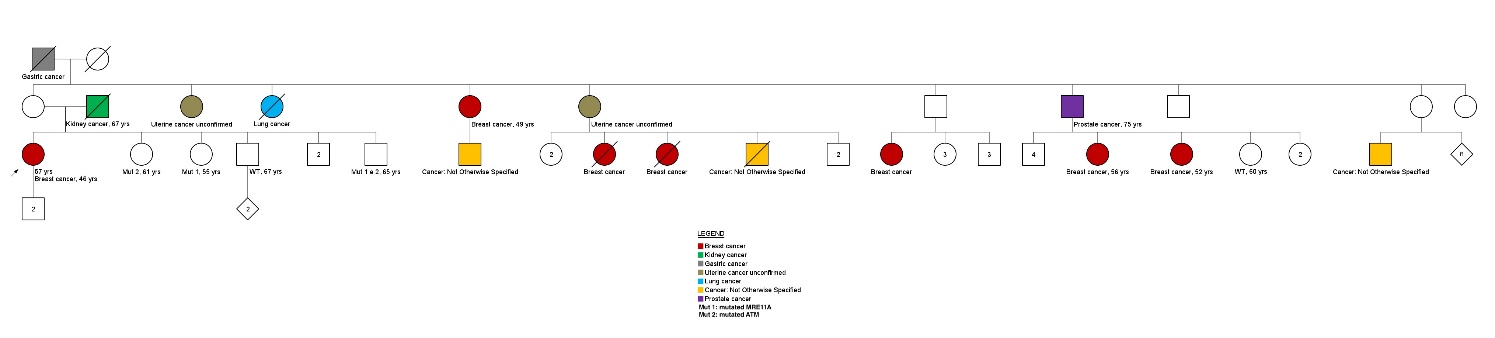
*

**Supplementary Figure 5:** Family history. Pedigree family ID133. Variant c.482A>G in the *MRE11A* and c.4709T>C in the *ATM.*

*
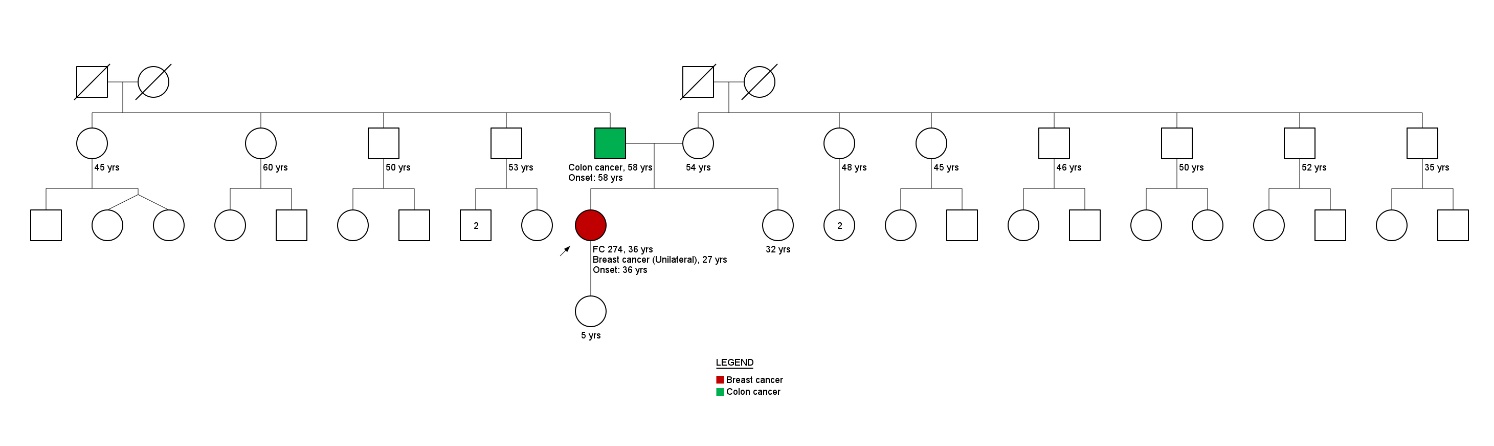
*

**Supplementary Figure 6:** Family history. Pedigree family ID274. Variant c.281T>C in the *RAD50.*

*
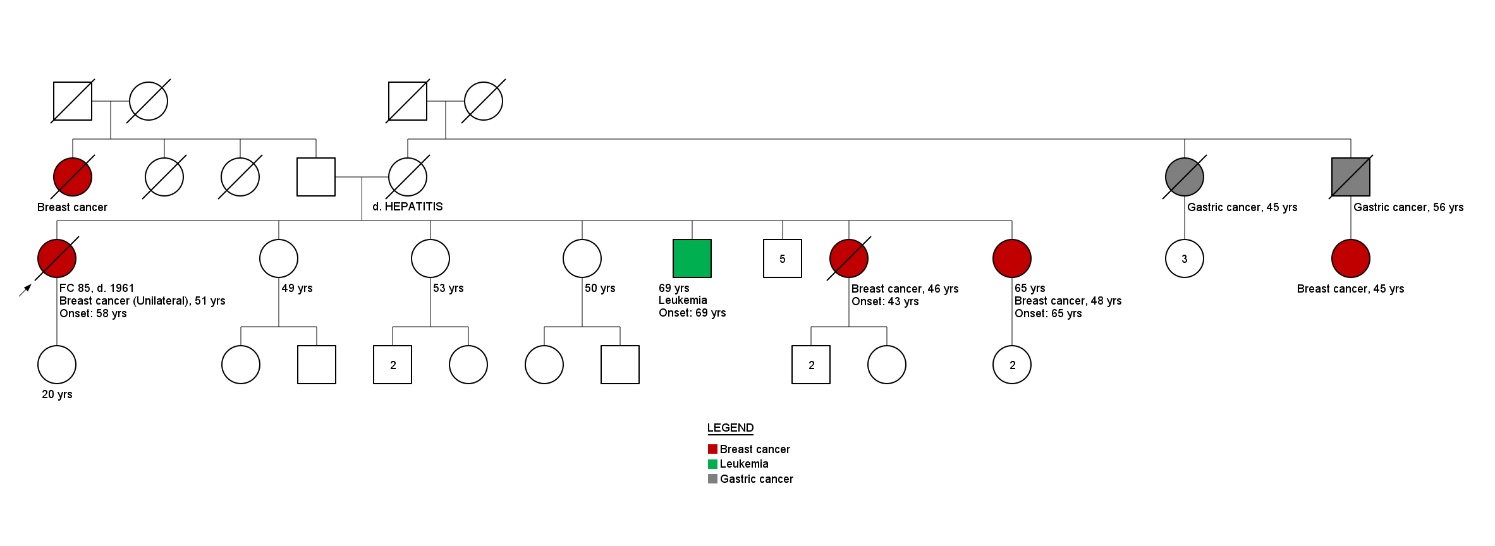
*

**Supplementary Figure 7:** Family history. Pedigree family ID85. Variant c.1810C>T in the *ATM* and c.1001A>G in *PALB2* gene.
